# Involvement of the ghrelin system in the maintenance and reinstatement of cocaine-motivated behaviors: a role of adrenergic action at peripheral β1 receptors

**DOI:** 10.1038/s41386-021-01249-2

**Published:** 2021-12-18

**Authors:** Zhi-Bing You, Ewa Galaj, Francisco Alén, Bin Wang, Guo-Hua Bi, Allamar R. Moore, Tristram Buck, Madeline Crissman, Sruti Pari, Zheng-Xiong Xi, Lorenzo Leggio, Roy A. Wise, Eliot L. Gardner

**Affiliations:** 1 Molecular Targets and Medications Discovery Branch, Intramural Research Program, National Institute on Drug Abuse, Baltimore, MD USA; 2 Behavioral Neuroscience Branch, Intramural Research Program, National Institute on Drug Abuse, Baltimore, MD USA; 3 Clinical Psychoneuroendocrinology and Neuropsychopharmacology Section, Translational Addiction Medicine Branch, Intramural Research Program, National Institute on Drug Abuse and National Institute on Alcohol Abuse and Alcoholism, Baltimore, MD USA; 4 Medication Development Program, Molecular Targets and Medications Discovery Branch, Intramural Research Program, National Institute on Drug Abuse, Baltimore, MD USA; 5 Present Address: Departamento de Psicobiología, Facultad de Psicología, Universidad Complutense de Madrid, Campus de Somosaguas, 28224 Madrid, Spain; 6 Present Address: McLean Hospital, Harvard University, Belmont, MA USA

**Keywords:** Motivation, Addiction, Peptide hormones, Reward

## Abstract

Cocaine addiction is a significant medical and public concern. Despite decades of research effort, development of pharmacotherapy for cocaine use disorder remains largely unsuccessful. This may be partially due to insufficient understanding of the complex biological mechanisms involved in the pathophysiology of this disorder. In the present study, we show that: (1) elevation of ghrelin by cocaine plays a critical role in maintenance of cocaine self-administration and cocaine-seeking motivated by cocaine-conditioned stimuli; (2) acquisition of cocaine-taking behavior is associated with the acquisition of stimulatory effects of cocaine by cocaine-conditioned stimuli on ghrelin secretion, and with an upregulation of ghrelin receptor mRNA levels in the ventral tegmental area (VTA); (3) blockade of ghrelin signaling by pretreatment with JMV2959, a selective ghrelin receptor antagonist, dose-dependently inhibits reinstatement of cocaine-seeking triggered by either cocaine or yohimbine in behaviorally extinguished animals with a history of cocaine self-administration; (4) JMV2959 pretreatment also inhibits brain stimulation reward (BSR) and cocaine-potentiated BSR maintained by optogenetic stimulation of VTA dopamine neurons in DAT-Cre mice; (5) blockade of peripheral adrenergic β1 receptors by atenolol potently attenuates the elevation in circulating ghrelin induced by cocaine and inhibits cocaine self-administration and cocaine reinstatement triggered by cocaine. These findings demonstrate that the endogenous ghrelin system plays an important role in cocaine-related addictive behaviors and suggest that manipulating and targeting this system may be viable for mitigating cocaine use disorder.

## Introduction

The addictive properties of cocaine relate to its ability to enhance dopamine (DA) transmission in the reward circuitry-crucially involving DA neurons originating from the substantia nigra and ventral tegmental area (VTA) [1, 2]. Chronic cocaine-induced adaptations within the DA system, as well as local or distal inputs to this system, are critical for development of the drug-seeking and drug-taking behaviors that characterize addiction [3–7]. Besides the neuronal input control, VTA DA neurons are sensitive to changes in metabolic state and respond to a variety of state regulators arising from peripheral sources, as feeding increases DA release with a greater magnitude in hungry versus sated animals [8, 9]. Congruently, changes in the actions of a variety of appetite-regulating hormones in the VTA modifies not only DA release but also behaviors associated with both food and rewarding drugs [10–13]. We have recently reported that both cocaine-taking and anticipation of cocaine in rats are associated with dysregulation of a variety of appetite-regulating hormones with a decline in anorexic and an elevation in orexigenic hormone levels in circulation, respectively [14]. However, the specific roles of most of these hormones in the rewarding and motivational effects of cocaine remain to be fully explored.

Ghrelin, a 28-amino acid orexigenic hormone, is most recognized for its role in stimulating growth hormone release and regulating feeding-related behaviors [15–18], via the growth hormone secretagogue receptor 1a (GHS-R1a, also termed ghrelin receptor [19, 20]). The post-translational acylation at the serine-3 residue, which is mediated by the membrane-bound enzyme ghrelin O-acyltransferase (GOAT) at the secretion sites, is essential for ghrelin to gain its binding property to GHS-R1a [19, 20]. Secreted ghrelin can be deactivated following degradation by either proteolysis into inactive fragments or by plasma esterase-mediated de-acylation to des-acyl ghrelin (DAG), the inactive form of ghrelin [21], and ghrelin action can be compromised by liver-expressed antimicrobial peptide 2 (LEAP2), a recently characterized endogenous GHS-R1a antagonist [22] that fluctuates in an opposite direction with ghrelin during either food deprivation or refeeding [23, 24]. Thus, it is likely that the dynamic signaling process of these ghrelin-related hormones may determine functional diversity of the ghrelin system.

The role of ghrelin in addiction has been more frequently studied in relation to alcohol use. Ghrelin levels in alcohol-dependent patients positively correlate with alcohol craving and risk of relapse [25, 26] (for a review, see: [27]). Exogenous ghrelin administration increases cue-induced alcohol craving [28] and alcohol self-administration [29] in alcohol-dependent heavy-drinking individuals, an observation consistent with several rodent studies [30] (for reviews, see: [30, 31]). With psychostimulants, ghrelin, administered either systemically or centrally, potentiates cocaine-induced locomotor activity and conditioned place preference (CPP) [32–34] whereas blockade of GHS-R1a significantly attenuates these effects [35–38]. The roles of ghrelin signaling in cocaine self-administration and reinstatement have not yet been explored.

The aim of the present study was to determine whether cocaine-motivated behaviors impact the function of the endogenous ghrelin system, and whether such an impact, if any, plays a role in cocaine-motivated behaviors. We first assessed the effects of cocaine self-administration, cocaine-seeking and yoked infusions of cocaine or cocaine methiodide (an enantiomer of cocaine that does not cross the blood-brain barrier) on fluctuations of plasma ghrelin, DAG and LEAP2 levels and the effects of acquisition of cocaine self-administration on GHS-R1a mRNA expression in VTA neurons. We next assessed the effects of GHS-R1a blockade by JMV2959 on cocaine self-administration, on cocaine-seeking either driven by the stimuli associated with cocaine self-administration or triggered by priming injection of cocaine or on brain stimulation reward (BSR) maintained by optogenetic stimulation of VTA DA neurons. Finally, as cocaine is a sympathomimetic and sympathetic action at β1 adrenergic receptors plays a major role in ghrelin secretion [39, 40], we assessed whether GHS-R1a blockade plays a role in reinstatement of cocaine-seeking triggered by yohimbine, a potent activator of the noradrenergic system, and whether there is an involvement of β1 receptors in cocaine-induced ghrelin elevation and cocaine-motivated behaviors following pretreatment with atenolol, a peripherally active β1 antagonist.

## Materials and methods

### Subjects

Male Long-Evans rats (Charles-River Laboratories, Raleigh, NC, USA.) were used for all experiments except BSR testing for which male DAT-Cre mice were used. Upon arrival, animals were group-housed in an animal facility under a reversed 12 h light-dark cycle (light on at 7:00 PM) with free access to food and water and allowed to acclimate to the new environment for at least 7 days prior to study initiation. Male DAT-Cre mice used in the BSR experiments were bred at the National Institute on Drug Abuse, Intramural Research Program (NIDA-IRP) using heterozygous animals and their genetic background has been reported previously [41]. All procedures were approved by the Animal Care and Use Committee of the National Institute on Drug Abuse (NIDA) and were consistent with the *Guide for the Care and Use of Laboratory Animals* (8th edition, National Research Council, 2011).

### Animal surgery

Rats (275–325 g) were surgically implanted with a micro-renathane intravenous catheter (Braintree Scientific Inc., Braintree, MA, USA) under ketamine and xylazine (90 and 10 mg/kg i.p., respectively) anesthesia according to procedures described previously [42]. After surgery, the catheters were flushed daily with a gentamicin–heparin–saline solution (0.1 mg/ml gentamicin and 30 IU/ml heparin, ICN Biochemicals, Cleveland, OH, USA) to prevent catheter clogging and infection. The animals were allowed to recover for at least 5 days before behavioral training started.

DAT-Cre mice (∼4 weeks of age) used in BSR testing were first anesthetized with ketamine and xylazine, followed by a stereotaxic injection of 150 nl of adeno-associated virus solution that carries channelrhodopsin-2 and enhanced green fluorescent protein (AAV-EF1a-DIO-ChR2-EGFP; ∼2 × 10^12^ genomes/ml, UNC Vector Core, University of North Carolina at Chapel Hill, NC, USA) bilaterally into the VTA using a micropump (Micro-4, World Precision Instrument, Sarasota, FL, USA) at a speed of 50 nL/min. The coordinates for VTA injection were AP + 3.28 mm, ML ± 1.2 mm, and DV − 4.48 mm inserted with a 10° angle toward the midline [43]. Following the virus injection, bilateral custom-made ferrule fibers (Inner diameter, 200 μm, Thorlabs, Newton, NJ, USA) were implanted 0.1 mm above the injection site. The mice were then allowed to recover for at least 4 weeks, to enable full AAV expression and ChR2 trafficking, before optical self-stimulation experiments began.

### Self-administration training

Cocaine self-administration training was conducted in an operant conditioning chamber (Med Associates Inc., Georgia, VT, USA) according to procedures described previously [44]. Briefly, following recovery from surgery, each rat was transported to the training room and allowed to lever-press for cocaine at a unit dose of 1 mg/kg/infusion under fixed ratio-1 (FR-1) reinforcement during a daily 3-hr session for 14 days. Saline-trained rats in experiment 1 were simply exposed to the same environment and their active lever-presses resulted in saline infusion. Animals used to test the effects of yoked drug infusions on hormone levels in Experiment 1 were trained under the same conditions but during a daily 4-hr session.

### BSR training

The procedure for BSR training was described in detail previously [41]. Briefly, following recovery from surgery, mice were trained to respond on the active lever in standard mouse operant chambers (Med Associates Inc.) for a 1‐s pulse train of laser stimulation at 473 nm wavelength (20 mW, 5 ms duration, 25 Hz) in daily, 1‐hr sessions. Inactive lever responses were recorded but had no scheduled consequences. After acquiring stable responding for 1‐week, mice were trained on a rate‐frequency program during which six stimulation frequencies (100, 50, 25, 10, 5, and 1 Hz) were available for self‐stimulation in descending order for 10‐min each. Once stable responding with < 20% variation across 3 consecutive sessions was established, the test phase began.

### Experiment 1: Ghrelin signaling in response to cocaine-motivated behaviors

To assess the effects of cocaine-motivated behaviors on ghrelin signaling, we measured the fluctuations of plasma ghrelin, DAG and LEAP2 levels in 8 cocaine-trained and 8 saline-trained rats following giving them an additional session after training. Another 8 cocaine-trained rats were tested following an extinction session. Blood samples (0.3 ml) were taken from each rat via the i.v. catheter that was used for drug or saline infusions immediately before the test session, 1 h into the session and at the end of session (3 hr). Samples were immediately transferred into EDTA coated tubes that contained p-hydroxymercuribenzoic acid in a final concentration of 1 mM to preserve ghrelin from degradation, and centrifuged at 4 ^o^C, 4000 *g* for 15 min. The supernatant from each sample was stored at −80 °C until they were assayed.

Ghrelin and DAG levels were assayed using ELISA kits from ALPCO (Salem, NH, USA). LEAP2 levels were assayed using ELISA kits from MyBioSource, Inc. (San Diego, CA, USA). Sample collection and storage, as well as hormone assays were performed following the manufacturer’s instructions. The intra- and inter-assay variations were less than 7 and 8% for ghrelin and DAG, 10 and 12% for LEAP2 respectively. Hormone levels were interpolated using the four-parameter logistic regression for standard curve fitting for each ELISA plate.

The fluctuations of plasma ghrelin and DAG were tested in 6 additional groups (3 cocaine-trained and 3 saline-trained) to assess the contributions of conditioned stimuli and cocaine’s peripheral action to the ghrelin responses. On the following day following completion of training, one group from each training condition were simply given another session under their training conditions. The remaining 2 groups from each training condition received unearned infusions of either cocaine or cocaine methiodide (1.3 mg/kg/infusion, the same molar concentration of 1 mg/kg cocaine), a synthetic cocaine analog that does not cross the blood-brain barrier [45], “yoked” to the earned infusions of an executive rat in the cocaine-self-administration group. Blood samples were collected at 0, 0.5, 2 and 4 h into the session, and processed as described above.

To assess the effects of cocaine self-administration on VTA GHS-R1a mRNA levels, eight additional rats were used – four trained for cocaine self-administration and another four trained for saline self-administration. On the day following completion of training, the animals were euthanized before the regular training session and the brains were extracted and stored as described previously [46]. GHS-R1a mRNA levels in VTA neurons were analyzed using RNAscope Multiplex Fluorescent Reagent kits according to the manufacturer’s instruction [Advanced Cell Diagnostics (ACD), Newark, CA, USA]. Briefly, immediately before brain slicing, each brain was placed on a cryostat (CM 3050 S) at −20 °C for 1 h for temperature equilibration and then coronal sections were cut at 16 μm thickness and mounted directly onto Super Frost Plus slides (Fisher, Cat. no. 12-550-15). The slide fixation, protease pretreatment, probe hybridization, signal preamplification and amplification, and fluorescent labeling steps were carried out according to the *User Manual for Fresh Frozen Tissue* (ACD, Inc). Four RNAscope probes – Ghsr1a (Cat. #431991), Slc6a3 (Cat. # 319621-C2), Slc32a1 (Cat. # 424541-C3) and Slc17a6 (Cat. # 317018-C3) – were used to detect GHS1a mRNA, DAT mRNA, and vGlut2 mRNA in the VTA, respectively (ACD Inc). Fluorescent images of labeled cells in the VTA were captured using a KeyenceX-BZ800 microscope. The mRNA signals in each individual cell were processed and quantified using Keyence Image Analyzer software.

### Experiment 2: GHS-R1a antagonism of cocaine-motivated behaviors

*JMV2959 on cocaine-taking and cocaine-seeking*. Four groups of rats (*n* = 8) were randomly assigned in these experiments following completion of the initial training sessions. Daily self-administration of cocaine continued until average drug infusions/session varied less than 10% over 3 consecutive sessions (14-18 sessions). On the test day, the 4 groups of rats were first systemically injected with either vehicle (saline) or a dose of JMV2959 (0.3, 3, or 6 mg/kg, i.p.). Cocaine self-administration testing began 15 min after the drug pretreatment. Following completion of the self-administration testing, the rats were allowed to self-administer cocaine for 4 additional sessions. On the following day, they were pretreated with either vehicle or one of the 3 doses of JMV2959. Fifteen minutes following JMV2959 pretreatment, the animals were tested in an extinction session during which saline was substituted for cocaine. Animals’ responses on the levers and drug or saline infusions during the 2 tests were recorded.

*JMV2959 on reinstatement of drug-seeking triggered by cocaine and yohimbine*. To assess whether ghrelin signaling plays a role in reinstatement, 7 groups of rats (*n* = 8 each) were first trained for cocaine self-administration as described above and then underwent extinction sessions. The extinction sessions were identical to the self-administration sessions except that animals’ responses on the active lever produced no scheduled consequences. Extinction sessions continued until the animals’ average active lever-presses/session decreased to less than 10 over 3 consecutive sessions. Reinstatement testing was performed on the day following the completion of extinction training. Four groups were first pretreated with either saline or one of the 3 doses of JMV2959 (0.3, 3 and 6 mg/kg, i.p.) 15 min before a cocaine challenge (10 mg/kg, i.p.). The remaining 3 groups were pretreated with either saline or one of the 2 high doses of JMV2959 before a yohimbine challenge (1.5 mg/kg, i.p.). The animals were allowed to lever-press for another extinction session immediately following cocaine or yohimbine challenge. Their responses on the active and inactive levers were assessed.

*JMV2959 on BSR maintained by optogenetic stimulation of VTA DA neurons.* To assess whether the ghrelin system plays a role in mesolimbic DA function, 7 mice were repeatedly pretreated with saline and two doses of JMV2959 (6, 12 mg/kg, i.p.) 15 min before the test session after training. The 3 tests were separated by 3 additional training sessions. Eight additional mice were used to assess the involvement of ghrelin in the potentiating effects of cocaine on BSR. They were pretreated with saline or JMV2959 (6 or 12 mg/kg, i.p.) 15 min prior to either saline or cocaine (4 mg/kg, i.p.) and their responses on the levers at various stimulation frequencies were measured during the test session.

### Experiment 3: Effects of atenolol on ghrelin levels and cocaine-motivated behaviors

Twenty-four cocaine-trained rats were divided into 4 groups (*n* = 6 each) following completion of cocaine self-administration training – 2 pretreated with atenolol (15 mg/kg, i.p.) and another two with vehicle (water). Thirty min later, one group from each pretreatment was challenged with an injection of cocaine (10 mg/kg, i.p.) and the remaining two groups with saline. Blood samples for ghrelin and DAG assay were collected immediately before cocaine or saline challenge (0 h), and 1 and 3 h following last injection. After blood collection, rats were redistributed into 3 groups and allowed to self-administer cocaine for 4 additional sessions and challenged on the next day with either vehicle or one of the two atenolol doses (5 or 15 mg/kg, i.p.) 30 min before a self-administration test session. Following completion of the self-administration test, the animals were put on extinction training as describe previously. Cocaine-triggered reinstatement test was performed on the following day after completion of the extinction training, 30 min after vehicle or atenolol pretreatment (5 or 15 mg/kg) and immediately after cocaine challenge. Animals’ responses on the levers were recorded.

### Drugs

Cocaine HCl, ketamine HCl and xylazine HCl were obtained from the NIDA-IRP research pharmacy. Cocaine methiodide was synthesized at NIDA-IRP. JMV2959 and atenolol HCl and yohimbine HCl were purchased from MilliporeSigma (St. Louis, MO, USA).

### Data analysis

All data were expressed as means ± SEM and were analyzed using one or two-way ANOVA, as appropriate. Significant main effects and interactions were followed by post hoc Student–Newman–Keuls tests for multiple group comparisons. Statistical analyses were performed using SigmaPlot 12 software (Systat Software, Inc., San Jose, CA, USA) and statistical significance was defined by *P* < *0.05*. Data from RNAscope analysis were analyzed using a two-tailed Student’s t-test.

## Results

### Ghrelin and DAG levels are robustly elevated by actual and anticipated cocaine

**Fig. 1:**
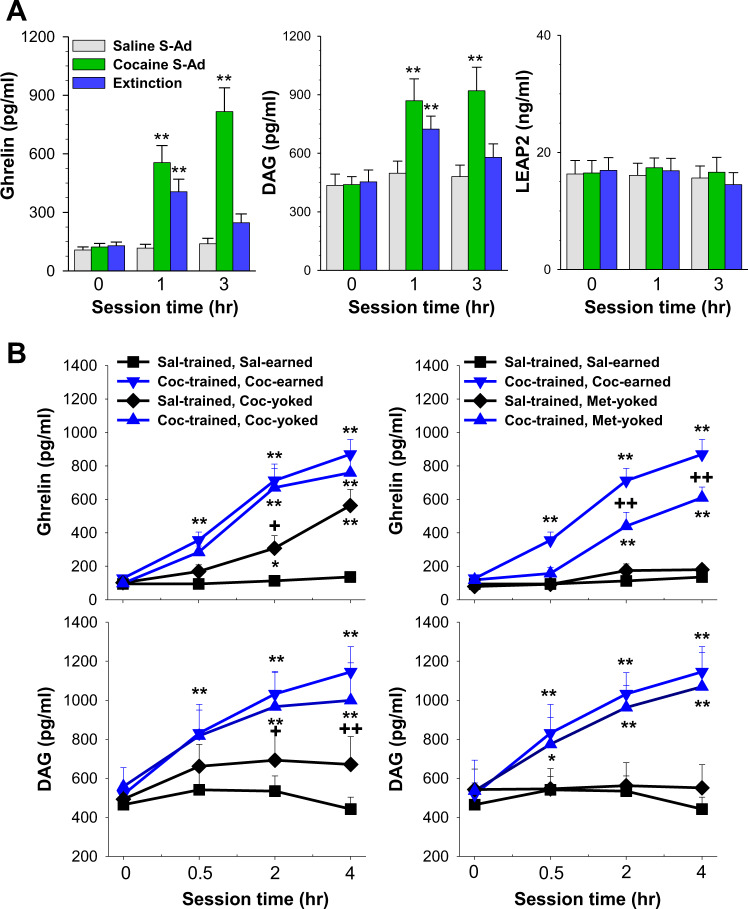
Plasma ghrelin and related hormone levels during cocaine or saline self-administration or extinction (saline substitution for cocaine, Fig. 1A) or during receipt of yoked infusions of cocaine or cocaine methiodide (Fig. 1B) following 14 days of cocaine or saline self-administration. Ghrelin and DAG levels rose significantly during cocaine self-administration or during an initial extinction session in cocaine-trained rats but not in saline-trained rats tested during a saline self-administration session. LEAP2 levels were not changed in either group. Both ghrelin and DAG were similarly elevated in cocaine-trained rats that self-administered or received yoked cocaine during the test session: Yoked cocaine also showed elevated ghrelin and DAG levels in saline-trained rats but at a significantly lower magnitude as compared to cocaine-trained rats. Yoked cocaine methiodide elevated ghrelin and DAG only in cocaine-trained rats but at lower magnitude than that seen following yoked cocaine (Fig. 1B). Asterisks, * and **, represent *P* < 0.05 and *P* < 0.01, respectively, as compared to pre-session levels (0 h); + and + +, *P* < 0.05 and *P* < 0.01, respectively, as compared to the corresponding values in the cocaine self-administration group. S-Ad, self-administration; Sal, saline; Coc, cocaine; Met, cocaine methiodide. *N* = 6-7 in each group.

Fig. 1A shows the fluctuations of mean (±SE) plasma ghrelin, DAG and LEAP2 levels in response to cocaine self-administration and extinction. Ghrelin and DAG levels were elevated during both self-administration and extinction testing. The elevations peaked at the end of the cocaine self-administration session while extinction responding resulted in significantly higher elevations of both ghrelin and DAG at 1 h into the test session. A two-way ANOVA for repeated measures over time revealed significant main effects of Time and Group and a significant Time x Group interaction for both ghrelin (Time, F_2, 15_ = 16.25, *P* < 0.001; Group, F_2, 30_ = 38.52, *P* < 0.001, Interaction, F_4, 30_ = 20.43, *P* < 0.001) and DAG (Time, F_2, 15_ = 4.22, *P* < 0.05; Group, F_2, 30_ = 26.69, *P* < 0.001, Interaction, F_4, 30_ = 7.65, *P* < 0.001). Neither cocaine self-administration nor extinction responding significantly altered plasma LEAP2 levels.

### Cocaine self-administration experience is associated with enhanced responses of ghrelin and DAG to cocaine and upregulation of VTA GHS-R1a mRNA expression

Yoked cocaine infusion significantly elevated plasma ghrelin and DAG levels in both cocaine- and saline-experienced rats with a significantly higher magnitude seen in the cocaine-experienced group (Fig. 1B, left panel). A two-way ANOVA revealed significant effects of Time, Group and Time x Group interaction for both ghrelin (Time, F_3, 23_ = 18.02, *P* < 0.0001; Group, F_3,69_ = 79.58, *P* < 0.0001, Interaction, F_9, 69_ = 10.39, *P* < 0.0001) and DAG (Time, F_3, 23_ = 3.61, *P* < 0.05; Group, F_3,69_ = 21.83, *P* < 0.0001, Interaction, F_9, 69_ = 4.10, *P* < 0.001). Yoked cocaine methiodide raised ghrelin and DAG only in the cocaine-experienced rats (Fig. 1B, right panel). A two-way ANOVA revealed significant effects of Time, Group and Time x Group interaction for both ghrelin (Time, F_3, 22_ = 34.84, *P* < 0.0001; Group, F_3,66_ = 71.96, *P* < 0.0001, Interaction, F_9, 66_ = 16.39, *P* < 0.0001) and DAG (Time, F_3, 22_ = 7.33, *P* < 0.01; Group, F_3,66_ = 18.65, *P* < 0.0001, Interaction, F_9, 66_ = 5.89, *P* < 0.0001).

**Fig. 2:**
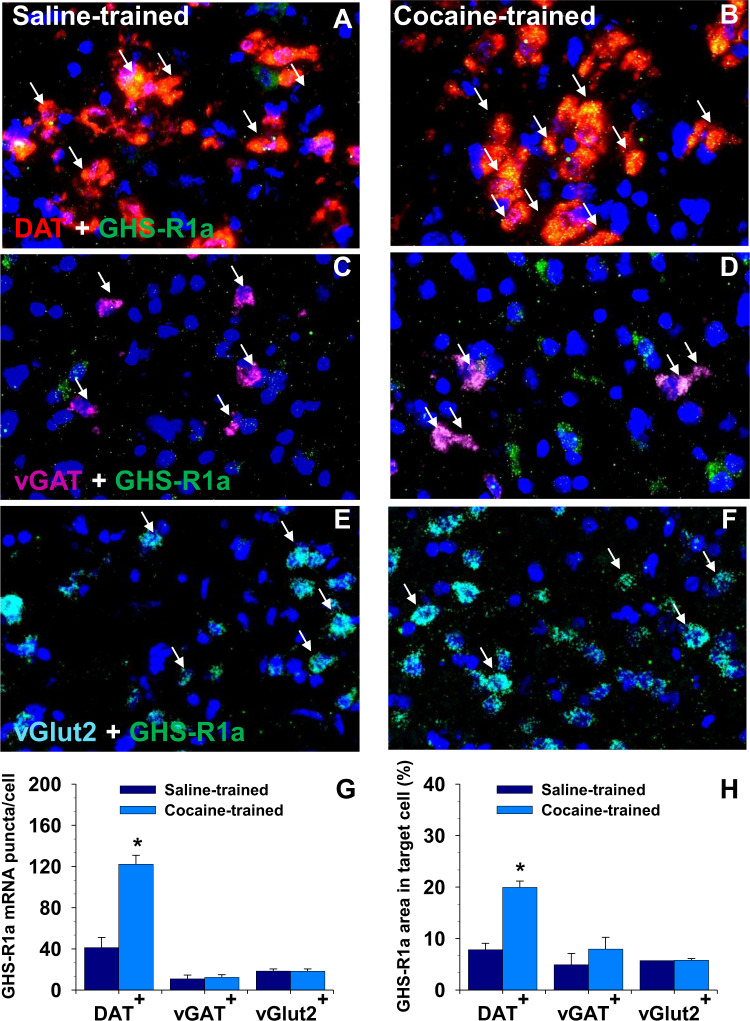
Acquisition of cocaine self-administration significantly upregulates GHS-R1a mRNA expression in rat VTA. Fig. 2A–F: Representative photomicrographs of VTA slice sections processed by multiplex RNAscope in situ hybridization for GHS-R1a, DAT, vGAT, and vGlut2. GHS-R1a signals were found in all three types of cells, but the upregulation is mostly restricted to DA neurons. Figure 2G, H: Quantitative analysis of GHS-R1a mRNA levels in VTA DA and GABA cells. Both GHS-R1a mRNA puncta and positive areas in DA but not GABA and glutamate cells are increased in the cocaine-trained rats. Arrows indicate dual-labeled neurons in the cocaine-trained and saline-trained rats. *, *P* < 0.0001 as compared to the saline-trained group. *N* = 4 in each group.

Acquisition of cocaine self-administration significantly elevated VTA GHS-R1a mRNA levels revealed by RNAscope in-situ hybridization analysis (Fig. 2). The elevation of GHS-R1a mRNA was mainly restricted to DA cells (Fig. 2A, B). Quantification analysis indicated that both the average numbers of GHS-R1a mRNA puncta (Fig. 2G) and GHS-1a signal area (Fig. 2H) in DA cells were significantly increased as compared to saline-experienced rats (Puncta/DA cell, *t* = 6.11, *P* < 0.0001; Area/DA cell, t = 9.99, *P* < 0.0001). No significant changes were observed in VTA GABA (Fig. 2C, D, G, H) and glutamate cells (Fig. 2E, F, G, H).

### GHS-R1a blockade by JMV2959 inhibits cocaine-taking, cocaine-seeking, and reinstatement of cocaine-seeking triggered by cocaine

**Fig. 3:**
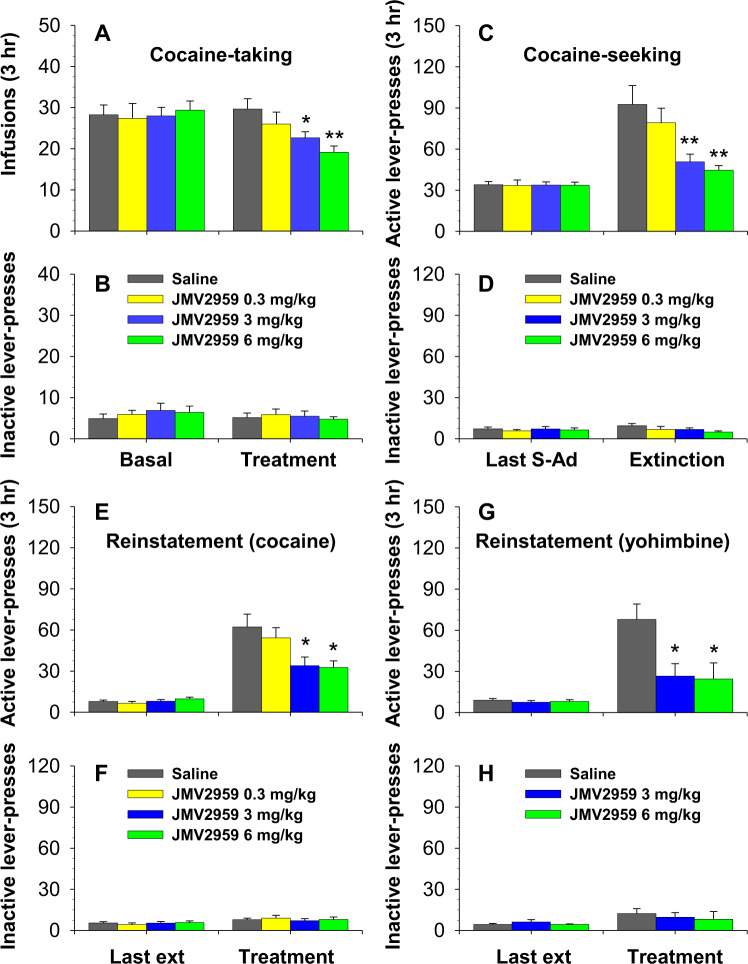
Effects of pretreatment with JMV2959 on cocaine self-administration, cocaine-seeking tested during an initial extinction session (saline substitution for cocaine) and reinstatement of cocaine-seeking triggered by cocaine or yohimbine. JMV2959 dose-dependently decreased cocaine infusions (Fig. 3A), active lever-presses for saline and cocaine-associated cues (Fig. 3C), and the active lever-presses triggered by cocaine (10 mg/kg, i.p.; Fig.3E) and yohimbine (1.5 mg/kg, i.p., Fig. 3G). JMV2959 pretreatment showed no effects on animals’ responding on the inactive lever (Figs. 3B, 3D, 3F, 3H). *, *P* < 0.05; **, *P* < 0.01 compared to saline group. *N* = 7–8 in each group.

Pretreatment with JMV2959 (0.3-6 mg/kg, i.p.) dose-dependently decreased cocaine self-administration as compared to either the last training session or the vehicle-pretreated group (Fig. 3A). A two-way ANOVA with session as a repeated measure revealed a significant effect of Group (F_2, 42_ = 4.82, *P* < 0.01) and a significant Group × Session interaction (F_3,42_ = 3.54, *P* < 0.05). A post hoc analysis indicated a significant reduction in cocaine-taking following pretreatment with either 3 or 6 mg/kg of JMV2959 as compared to the vehicle-pretreated group. Active lever-presses changed in a similar manner as cocaine infusions following pretreatment (data not shown). Pretreatment with JMV2959 produced no significant effects on inactive lever-presses (Fig. 3B).

**Fig. 4:**
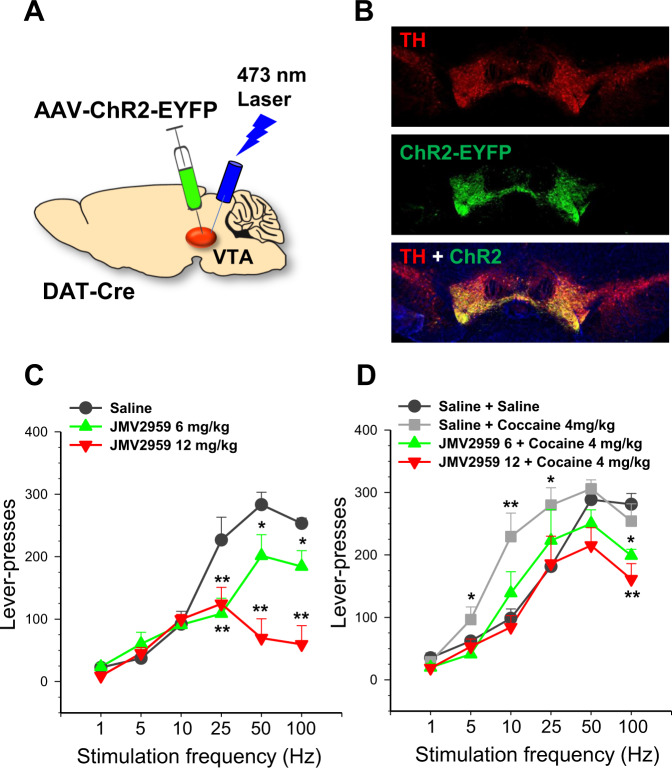
Effects of pretreatment with JMV2959 on BSR maintained by optogenetic self-stimulation of VTA DA neurons and on the potentiating effects of cocaine on BSR in DAT-Cre mice. A: A schematic diagram of the AAV‐ChR2‐eYFP microinjection and intracranial optical fiber implantation into VTA of DTA-Cre mice. B: Representative images indicating the expression of TH (up panel, red) and AAV‐ChR2‐EYFP (middle panel, green) and their colocalization (bottom panel, orange) in the VTA. C: JMV2959 pretreatment dose‐dependently shifted the stimulation frequency-response curve rightward as compared to saline pretreatment. D: JMV2959 pretreatment dose-dependently blocked the left shift of the frequency-response curve induced by cocaine. Coc, cocaine. *, *P* < *0.05*, **, *P* < *0.01*, as compared to vehicle treatment. *N* = 7-8 in each treatment.

When saline was substituted for cocaine, JMV2959 pretreatment (0.3-6 mg/kg, i.p.) significantly inhibited cocaine-seeking as demonstrated by selective decreases in animals’ responding on the active lever (Fig. 3C). A one-way ANOVA revealed a significant treatment effect on active lever-presses (F_1,42_ = 35.60, *P* < 0.0001) but not on inactive lever-presses (Fig. 4D; *P* = 0.49). Post hoc analysis revealed significant reduction in active lever-presses in groups pretreated with either 3 or 6 mg/kg of JMV2959 as compared to saline controls.

A priming injection of either cocaine (10 mg/kg, i.p.) or yohimbine (1.5 mg/kg, i.p.) robustly reinstated active lever-presses in the vehicle-pretreated group. These reinstatements were dose-dependently attenuated by JMV2959 pretreatment (Fig. 3E, G). A one-way ANOVA revealed a significant Group effect for both cocaine-induced reinstatement (F_3,38_ = 4.38, *P* < *0.02*) and yohimbine-induced reinstatement (F_2,18_ = 5.86, *P* < *0.02*) groups. Either JMV2959 or yohimbine pretreatment showed no effects on animals’ inactive lever-presses (Fig. 3F, H).

### GHS-R1a blockade by JMV2959 inhibits BSR maintained by optogenetic stimulation of VTA DA neurons

Pretreatment with JMV2959 (6, 12 mg/kg, i.p.) significantly inhibited optogenetic self-stimulation of VTA DA neurons in DAT-Cre mice as demonstrated by a dose-dependent rightward-shift of the stimulation frequency-response curve following JMV2959 pretreatment (Fig. 4C). A two-way ANOVA revealed significant main effects of Treatment (F_2, 12_ = 10.82, *P* < 0.01), and Stimulation Frequency (F_5,30_ = 41.76, *P* < 0.0001) and a significant interaction of Treatment x Stimulation Frequency on active lever-presses (F_10,60_ = 9.30, *P* < 0.0001). Post-hoc analysis revealed a significant decrease in responses on the active lever at 25–100 Hz following pretreatment with either dose of JMV2959. Cocaine (4 mg/kg, i.p.) enhanced lever-presses for BSR by shifting the stimulation frequency-response curve leftward (F_5,35_ = 9.30, *P* < 0.0001). Pretreatment with JMV2959 dose-dependently blocked the leftward shift of the stimulation frequency-response curve induced by cocaine (Fig. 4D). A two-way ANOVA with Treatment and Stimulation Frequency as repeated measures revealed significant main effects of Treatment (F_3,18_ = 5.05, *P* < *0.02*) and Frequency (F_5,30_ = 107.3, *P* < 0.0001) and Treatment x Stimulation Frequency interaction (F_15,90_ = 1.78, *P* < 0.05).

### Atenolol dose-dependently inhibits cocaine-induced increase in ghrelin levels and inhibits cocaine self-administration and reinstatement of cocaine-seeking

**Fig. 5:**
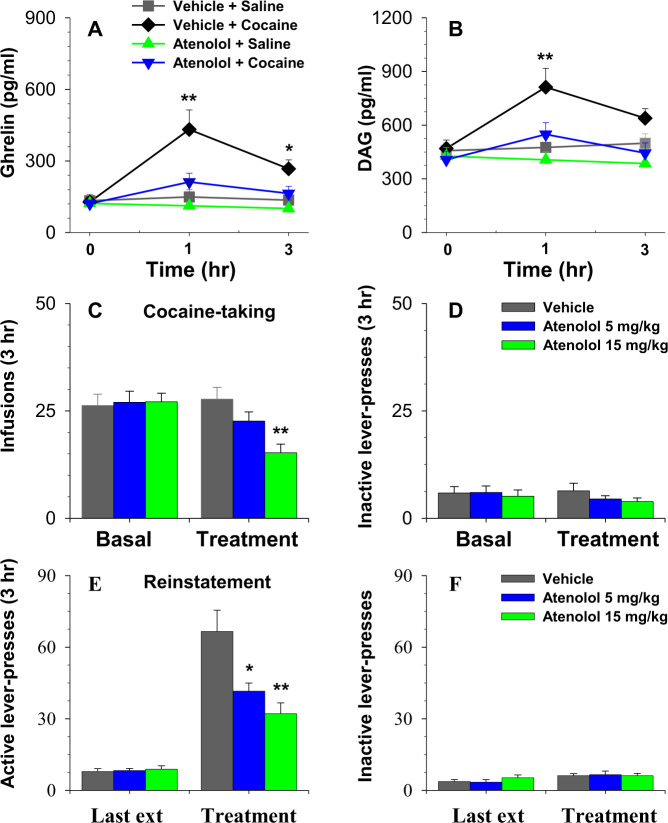
Effects of pretreatment with atenolol on plasma ghrelin and DAG levels, cocaine self-administration and reinstatement of cocaine-seeking. Atenolol (15 mg/kg, i.p.) potently attenuated the elevations in ghrelin and DAG levels induced by cocaine (10 mg/kg, i.p.; Fig. 5A, B). Atenolol pretreatment (5-15 mg/kg) dose-dependently reduced cocaine infusion (Fig. 5C) and the active lever-presses triggered by cocaine (10 mg/kg, i.p.). Atenolol showed no effects on animals’ responding on the inactive lever (Fig. 5D, F). *, *P* < 0.05; **, *P* < 0.01 as compared to vehicle group. #, *P* < 0.01 compared to corresponding basal levels. *N* = 6–8 in each group.

Plasma ghrelin and DAG levels were significantly elevated one hour following a systemic injection of cocaine (10 mg/kg, i.p.) in the vehicle-pretreated group. Atenolol pretreatment (15 mg/kg, i.p.) significantly attenuated these elevations induced by cocaine (Fig. 5A, B). ANOVA revealed a significant main effect of Time for ghrelin (F_2, 15_ = 6.21, *P* < 0.02). ANOVA also revealed significant main effects of Group and Time x Group interactions for both ghrelin (Group, F_3, 45_ = 4.49, *P* < 0.01, Interaction, F_6, 45_ = 11.22, *P* < 0.001) and DAG (Group, F_3, 45_ = 3.78, *P* < 0.02, Interaction, F_6, 45_ = 10.74, *P* < 0.001).

Pretreatment of rats with atenolol (5-15 mg/kg, i.p.) dose-dependently inhibited responding on the active lever (data not shown) and cocaine infusions during a self-administration test session (Fig. 5C). A two-way ANOVA revealed significant effects of Time (F _1,14_ = 4.62, *P* < 0.05), Group (F_2,28_ = 5.36, *P* < 0.02) and Time x Group interaction (F _2,28_ = 4.45, *P* < 0.05).

Pretreatment with atenolol (5–15 mg/kg, i.p.) dose-dependently inhibited the reinstatement of active lever-presses triggered by cocaine (Fig. 5E). ANOVA revealed a significant effect of Group on active lever-presses (F_2,19_ = 8.90, *P* < 0.01). Pretreatment with atenolol showed no effects on inactive lever-presses tested under either condition (Fig. 5D, F).

## Discussion

Elevation of endogenous ghrelin signaling by cocaine represents a potential mechanism by which cocaine and cocaine-associated stimuli reinforce drug-taking and motivate drug-seeking in cocaine-experienced rats. Specifically, cocaine self-administration and cocaine-seeking behavior each robustly elevates circulating ghrelin levels. Atenolol’s potent inhibition of cocaine-induced ghrelin elevation suggests an involvement of a peripheral adrenergic action in cocaine-related effects. Further, acquisition of cocaine self-administration is associated with an upregulation of GHS-R1a mRNA levels in the VTA, a brain region crucial for cocaine reward. The GHS-R1a mRNA upregulation is primarily restricted to DA neurons, which indicates that cocaine-induced ghrelin signaling enhancement could occur in the mesolimbic reward system via either alterations in ghrelin secretion, receptor signaling, or both. Behaviorally, GHS-R1a blockade dose-dependently inhibits cocaine self-administration and cocaine-seeking, as well as relapse to cocaine-seeking triggered by cocaine. GHS-R1a blockade also potently inhibits BSR maintained by optogenetic stimulation of VTA DA neurons and inhibits the potentiating effects of cocaine on BSR. Further, we show that atenolol, at a dose that inhibits the effects of cocaine on ghrelin, also potently inhibits cocaine self-administration and cocaine-trigged relapse to cocaine-seeking. These findings suggest the endogenous ghrelin system as a critical substrate by which cocaine’s activation of the peripheral adrenergic system is centrally conveyed and cocaine-motivated behaviors are modulated.

Unlike for ghrelin and DAG, the plasma levels of the endogenous ghrelin antagonist LEAP2 were not affected by either cocaine self-administration or cocaine-seeking. In contrast to ghrelin, LEAP2 levels decrease in response to fasting and increase following refeeding, and LEAP2 given in vivo inhibits ghrelin-induced food intake [22, 47]. Circulating LEAP2 and ghrelin inversely correlate with body mass, indicating that they act synergistically in response to energy demand [23, 48, 49]. Thus, the LEAP2-ghrelin circulation ratio has been proposed as a means of assessing ghrelin function [23]. If true, the enhanced ghrelin signaling observed in the present study may well have derived mainly from elevated ghrelin. The lack of LEAP2 response suggests that the secretion of these two hormones can also be disassociated under certain circumstances, such as cocaine exposure.

The present data reveal both unconditioned and conditioned elevations of ghrelin levels in rats regularly self-administering cocaine. This is shown by the ghrelin elevations in cocaine-trained rats receiving an unexpected extinction test and in rats receiving yoked cocaine and cocaine methiodide infusions. While the elevations of ghrelin caused by yoked cocaine in the saline-trained rats may reflect a simple stimulatory effect of cocaine, the significantly higher response of ghrelin in the cocaine-yoked, cocaine-trained rats and the elevations of ghrelin during the extinction session suggest that acquisition of cocaine self-administration is associated with acquisition of the stimulatory properties on ghrelin by cocaine conditioned stimuli. Compared to cocaine, yoked cocaine methiodide caused significantly lower ghrelin responses in the cocaine-trained rats and showed no effects in the saline-trained rats. Congruent with a conditioned role for cocaine in ghrelin secretion, circulating ghrelin levels in rats has been shown to positively correlate with cue-triggered cocaine-seeking [50]. Together, while these findings indicate a conditioned effect of cocaine, they suggest that the unconditioned effect of cocaine on ghrelin requires cocaine acting centrally.

The stimulatory effects of cocaine on ghrelin appear to be mediated by adrenergic action at β1 receptors, as pretreatment with atenolol potently attenuated cocaine-induced increases in ghrelin and DAG levels. Circulating ghrelin is derived predominantly from endocrine cells in the stomach, where ghrelin secretion is under major control by adrenergic sympathetic inputs [39, 51, 52] mediated by β1 adrenergic receptors [40]. Local administration of noradrenaline or adrenaline to this region increases extracellular ghrelin levels as assessed by microdialysis, and noradrenaline also potently stimulates ghrelin secretion from ghrelin cells in vitro [53, 54] Cocaine is a nonselective monoamine uptake inhibitor that increases noradrenergic transmission through blockade of presynaptic noradrenaline reuptake. Cocaine is also a stressor that elevates circulating noradrenaline and adrenaline levels [55]. The inhibitory effects of atenolol seen in the present study suggest that cocaine may increase ghrelin secretion through activation of both synaptic and non-synaptic adrenergic signaling in ghrelin cells. Intriguingly, β1 adrenergic receptor antagonism has also been shown to attenuate both pre-prandial and stress-induced enhancement of ghrelin, indicating that sympathetic activation is likely a common process by which cocaine, nutrients and stress control ghrelin release. This conclusion is consistent with our previous behavioral studies in which cocaine at the same molar dose reinstated cocaine-seeking more potently than cocaine methiodide [45].

The present findings do not exclude other mechanisms by which cocaine can regulate ghrelin levels. In contrast to ghrelin, cocaine self-administration progressively inhibits the levels of several anorexic hormones including insulin and leptin [14]. Whether elevated ghrelin involves disinhibition caused by decreased insulin remains to be explored. Additionally, both cocaine and ghrelin are powerful activators of the hypothalamic-pituitary axis (HPA; [56–58]), including in individuals showing addictive behaviors [59–61], and HPA appears to serve as an inhibitory feedback system, as both central corticotropin releasing factor agonist and systemic glucocorticoid administrations significantly inhibit ghrelin secretion, while adrenalectomy-induced elimination of corticoids potentiates fasting-induced ghrelin elevation, an effect that can be normalized by glucocorticoid replacement [62–64].

The inhibitory effects of JMV2959 on cocaine self-administration and cocaine-seeking indicate a potential role for the ghrelin system in the maintenance of behaviors driven by cocaine and by conditioned reinforcers known to prolong drug-motivated behaviors [65]. Such maintenance is likely achieved, at least in part, by the action of the ghrelin system on the mesolimbic DA reward system. Unlike food and other natural rewards that stimulate DA release directly [66], cocaine activates DA transmission by inhibiting DA reuptake, a process that also inhibits DA impulse flow [67]. Importantly, maintenance of DA neuronal impulse flow is critical in cocaine reward. Given the important roles of ghrelin in stimulating VTA DA cell firing [68] and DA release in terminal regions [12], ghrelin elevation by cocaine or cocaine-associated stimuli may counteract the inhibitory effects of cocaine on DA impulse flow, and contribute to cocaine-elevated DA transmission and consequently to cocaine-motivated behaviors. Supporting this notion are findings that ghrelin given either systemically or locally into the VTA potentiates DA release and behaviors induced by cocaine [69–71]. However, this role of ghrelin would need sufficient peripheral ghrelin reaching brain regions involved in mediating reward. Peripheral ghrelin has been recently shown to cross both blood-cerebrospinal fluid (CSF) and blood-brain barriers, although at low levels [72, 73]. Blockade of GHS-R1a in VTA significantly inhibits drug-seeking in food-restricted but not normally fed rats [74]. These findings suggest that elevated ghrelin levels, as also seen in the present study, may have a centrally functional significance. This interpretation, however, doesn’t rule out an involvement of peripheral ghrelin or ghrelin in other brain regions in cocaine-motivated behaviors seen in the present study as peripheral ghrelin directly acts on hypothalamic neuropeptide Y (NPY) neurons that project to both nucleus accumbens (NAS) and VTA [75–77], and activation of these neurons significantly increases DA signaling in the NAS [77]. Central NPY administration increases the motivational effects of both drug and natural reward [78–80]. Further, in addition to VTA and hypothalamus, GHS-R1a is also densely located in the amygdala, dorsal raphé [81–83], and hippocampus where ghrelin elevates anxiety-like behaviors in rats [84–86] and increases the response of the amygdala to alcohol cues in heavy-drinking, alcohol-dependent individuals following i.v. ghrelin [29]. Therefore, the observed behavioral effects in the present study may result from both central and peripheral action of ghrelin and involve brain regions that mediate both positive and negative aspects of drug reinforcement [4, 6]. Additionally, GHS-R1a are found in VTA DA, GABA, and glutamate neurons and are likely upregulated in DA neurons following acquisition of the cocaine-taking habit as indicated by the significant increase in GHS-R1a mRNA levels in DA neurons in the present study. GSH-R1a is known to heterodimerize with both DA D1 and D2 receptors to potentiate DA action at these receptors [87–89]. Future studies are necessary to verify whether GHS-R1a heterodimerization is involved in the behavioral effects of JMV2959 found in the present study. GHS-R1a blockade by JMV2959 in the present study dose-dependently inhibited BSR and blocked the potentiating effects of cocaine on such behavior in DTA-Cre mice. We have found, in a parallel study, that JMV2959 at the similar dose range shows no effects on BSR maintained by VTA stimulation of NAS GABA neurons to this region in vGAT-Cre mice [90]. This finding while ruling out a potential motoric side effect of JMV2959, suggests that GHS-R1a signaling at the targeted neurons may be mainly responsible for the attenuation of reward stimulating behaviors seen in DAT-Cre mice, as GHS-R1a are not reliably detected in most forebrain regions including the NAS [91].

The findings that atenolol significantly inhibits cocaine self-administration and cocaine-induced elevations in ghrelin suggest that ghrelin may be a potential substrate by which the peripheral adrenergic system modulates cocaine-driven behaviors. The inhibitory effects of atenolol on cocaine self-administration are consistent with previous findings tested under similar experimental conditions [92]. Atenolol, as a β1 blocker mainly active in the periphery [93], potently attenuates cocaine-induced tachycardia [94], a sympathetic activation-mediated somatic sign normally seen in cocaine dependency during exposure to cocaine cues [95, 96], and cocaine withdrawal-induced anxiety [97]. However, the circuits that connect such action of atenolol with relevant central sites remain unclear. Interestingly, ghrelin given directly to the hippocampus, amygdala, or dorsal raphe nucleus elevates anxiety-like behaviors in rats and stress-induced elevation of circulating ghrelin is sufficient and necessary for stress-associated vulnerability to exacerbated fear learning [84–86]. Here, we show that yoked cocaine methiodide elevates circulating ghrelin only in cocaine-trained rats. Further, we have previously shown that systemic cocaine methiodide injection is sufficient to elevate glutamate and DA release in the VTA and to reinstate cocaine-seeking in cocaine-trained rats following behavioral extinction [45]. Ghrelin signaling in catecholaminergic neurons is involved in stress-induced food-reward [98].

The present study suggests that elevation of circulating ghrelin constitutes a process by which cocaine triggers drug-seeking. We show that targeting ghrelin signaling, directly via GHS-R1a blockade (by JMV2959) or indirectly via peripheral β1 receptor blockade (by atenolol) dose-dependently inhibits cocaine-triggered reinstatement of drug-seeking behavior in cocaine-trained rats after subsequent extinction of cocaine-taking behavior. Atenolol significantly inhibits the ghrelin elevation induced by cocaine. Further, we show that blockade of GHS-R1a effectively attenuates the reinstatement of cocaine-seeking triggered by yohimbine. Of note, yohimbine is a potent stressor and activator of the adrenergic system, including in animal models of addiction and in humans living with addictions [99–101]. Therefore, whether endogenous ghrelin represents one of the signaling systems that carry peripheral adrenergic signals to brain reward and stress regions is an interesting subject for future research. This suggests that apart from its primary direct action on DA, cocaine may also trigger drug-seeking through activation of an autonomic adrenergic system. Stress and adrenergic activation are each recognized as critical triggers in escalating prolonged access drug-taking [6, 102] and in relapse [103, 104]. Such effects are believed to result from the direct central actions of drugs and stressors [105]. However, given the positive correlation of noradrenergic activation with stress- and cue-induced craving [96, 106] and the significant roles of both ghrelin and adrenergic action reported here, ghrelin and the peripheral adrenergic system are likely to be mediators by which cocaine and stress act to control drug-motivated behaviors. Future studies are necessary to test whether atenolol modulates self-administration maintained by other addictive drugs and the reinstatement of drug-seeking behaviors caused by other stressors and drug-associated environmental cues.

The present study reveals a reciprocal stimulatory interaction between endogenous ghrelin signaling and behaviors caused by cocaine and cocaine-predictive cues with an involvement of peripheral β1 adrenergic action in such interaction. The stimulatory responses of ghrelin and DAG to cocaine self-administration seen in this study are similar to the previously reported responses of total ghrelin to 3,4-methylenedioxymethamphetamine or methamphetamine, two other psychostimulants, measured at varying time points following an acute systemic challenge in rats [107, 108]. Notably, although we also observed a significant elevation of ghrelin and DAG following i.p. injection of cocaine in cocaine-trained rats, we previously did not find such response in ghrelin in human subjects measured 2 hr following an i.v. cocaine challenge [109]. Whether this discrepancy is associated with difference in cocaine dose or route of administration, in the time exposure to cocaine (chronic vs. acute), or reflects a species-specific response remains to be determined. Furthermore, it is important to note that, in the present study, the reciprocal stimulatory interaction between endogenous ghrelin signaling and behaviors produced by cocaine and cocaine-predictive cues are demonstrated only in male rats. Given that sex differences may play a role in addictive behaviors and estrogens play a critical role in such propensity [110–113], future studies are necessary to assess whether such behavioral effects are generalizable to females and/or whether sexual differences exit. The behavioral findings in the present study are in concert with a role for ghrelin in cocaine reward and motivation reported from studies using other behavioral models such as CPP and locomotor sensitization [34, 38, 114, 115] and consistent with a general view that drug use behaviors are under the control of some of the same biological substrates as are involved in natural reward processing [5, 116, 117]. Therefore, manipulating and targeting this system may be viable for developing new treatments for cocaine use disorder.
